# Patients’ Perspective in Hereditary Ataxia

**DOI:** 10.1007/s12311-022-01505-1

**Published:** 2022-12-16

**Authors:** Sorina Gorcenco, Christin Karremo, Andreas Puschmann

**Affiliations:** Neurology, Department of Clinical Sciences Lund, Lund University, Skåne University Hospital, Getingevägen 4, 22185 Lund, Sweden

**Keywords:** Hereditary ataxia, Neurodegenerative disorders, Patients, Quality of life, Questionnaire

## Abstract

Hereditary ataxia represents a heterogeneous group of rare disorders with the chronic progression of motor symptoms that often become debilitating. Many forms include additional neurological, cognitive, or other symptoms. Most of these disorders lack specific treatment. We aimed to investigate aspects of patients’ quality of life, experiences, and expectations. Patients with a diagnosis of hereditary ataxia were identified from our center’s diagnostic register, direct referrals, and from a patient organization. We designed a questionnaire with 32 multiple-choice or open-ended questions on disability and impairment of daily life activities, the perceived effect of symptomatic and supportive therapies, coping strategies, and how they used and experienced various sources of information about their neurological disease. We also included the EQ-5D-3L quality-of-life instrument. Results were analyzed statistically for gender, age, and groups with and without a genetic diagnosis, and were compared to published data from the general population. Seventy-five patients returned the questionnaire. Patients reported considerable disease-related disability and impairment and had significantly lower quality-of-life scores than the general population. Physiotherapy and support from family or friends were important for patients’ overall well-being. Patients with a genetic diagnosis had a lower average age at onset and felt more well-informed about their disease than patients without a genetic diagnosis. Patients used internet sources but relied primarily on their doctors to obtain information about their disease. Our study provides insights into hereditary ataxia patients’ experiences that can lead to improvements in medical and nursing care for these patients.

**Supplementary Information:**

The online version contains supplementary material available at 10.1007/s12311-022-01505-1.

## Introduction

Hereditary ataxia comprises a large and complex group of clinically and genetically heterogeneous disorders that are characterized by gait impairment, incoordination of eye and limb movements, and dysarthria. The disease progression and survival are, however, variable between and within different types of hereditary ataxia. Some of the most important factors that can influence the disease severity and progression seem to be the age at onset and, for repeat expansion disorders, the length of the expansions. [1, 2] Other neurological symptoms such as polyneuropathy, hearing loss, or cognitive impairment may be associated with the progression of the ataxias. Such additional symptoms are often distinctive for a particular disease and, therefore, useful in confirming a diagnosis. The symptoms are generally chronically progressive and, for many patients, become debilitating [3–5]. For the majority of patients with hereditary ataxia, there is currently no specific or causative treatment.

Progressive deterioration of patients with hereditary ataxia has a major impact on the health-related quality of life. However, the number of studies assessing the quality of life and the patient-reported outcome is limited [6–8]. Studies of diverse other neurodegenerative diseases have shown that the subjective perception of health can be multifactorial and thus not only disease-related [9]. Therefore, we decided to further investigate the ataxia patients’ quality of life and how it compares to the general population, how patients perceive the diagnosis-related information, and where they find this information. We aimed to examine differences between the patients who have an established genetic diagnosis and the ones who do not. We also wanted to find out about what patients find to improve their well-being. The overarching aim was to explore if there is anything we can do better as clinicians to improve the care for ataxia patients.

## Patients and Methods

This study is based on patients enrolled in our ongoing NEUGEN-ataxia study. Adult patients with a diagnosis of hereditary ataxia (ICD-10 version: 2019 (who.int.) G11.0, G11.1, G11.2, G11.3, G11.8, or G11.9) were identified in the diagnosis register of the department of neurology at Skåne University Hospital with campuses in Lund (patient contacts from January 2011 to April 2020) and in Malmö (from January 2016 to April 2020), or were recruited through direct referrals from other neurologists, through their families, and the Swedish patient organization SCA-Network. Some of the patients had a genetic diagnosis of autosomal dominant or autosomal recessive ataxia at the time of inclusion in the study, but many patients did not have an exact diagnosis yet, thus blood samples were collected from all the patients with chronically progressive ataxia for genetic testing within the study (Supplementary Table 1).

All patients were systematically interviewed by the authors using open questions and standardized checklists for data extraction from clinical neurology records and on family history, imaging, and genetic examination. Clinical examination was performed by one of the authors using a standardized study protocol for the examination of ataxia patients, which included a detailed neurological examination with a special focus on speech, eye movements, coordination, and gait. The severity of the disease was measured by using the scale for the assessment and rating of ataxia (SARA) [10].

To assess aspects of the patient’s perspective and subjective quality of life, we designed a questionnaire containing 32 “multiple-choice” or “open-ended” questions. The questionnaire was distributed in paper format together with the study information or invitation letter and was filled by the patient or, in some cases, by their caretakers. The questions focused mainly on the patient’s perception of aspects of their well-being, their limitations in daily life activities, their reception of disease-related medical information, supportive care, and coping strategies. The questionnaire was created and used in the Swedish language; the authors’ English translation of the questionnaire is provided as Supplemental Material 1.

To test the subjective health status, we used EQ-5D-3L, a standardized instrument for assessing the quality of life with the following five domains: (i) mobility, (ii) self-care, (iii) usual activities, (iv) pain and discomfort, and (v) anxiety and depression. A request to use EQ-5D-3L for the study was registered and approved at the EuroQol website (https://euroqol.org) with the registration ID: 51,354. EQ-5D-3L is not disease-specific and allows the comparison of a patient group to the general population [11]. We chose EQ-5D-3L as it has been used previously in ataxia patients [6]. Furthermore, we wanted to compare the results of EQ-5D-3L from our patient group with the results from a sample of 534 Swedish citizens that underwent the testing through a self-applied postal survey and were randomly selected from the general population in a study from 1994. The used statistical method for comparison was Mann–Whitney *U* statistical test [12, 13].

Questionnaire responses were collected and stored digitally in SUNET Survey, a secure online tool available at Lund University, for easier data retrieval. Statistical analyses such as descriptive statistics, Pearson chi-square, and independent samples *t*-test were performed using the SPSS program.

## Results

### Study Population

A total of 158 patients with a diagnosis of hereditary ataxia were identified from the hospital and outpatient clinic diagnosis registers. Of these, a study information/invitation letter was sent to 96 who were alive at the start of our study and who had not received an alternative diagnosis. The letter was also sent to an additional 48 patients who were recruited through referrals, contact with families, and through the patient organization.

Five patients with Friedreich ataxia were excluded because they had been enrolled in a separate study at our department for ethical reasons to avoid overburdening patients with research studies. Four patients were excluded because clinical examination within this study indicated another diagnosis. We also excluded patients who resided outside our hospital’s geographical uptake area. In total, we included 88 patients with the diagnosis of hereditary ataxia.

The questionnaire was returned by 75 patients (85.2%). Characteristics of the studied population are illustrated in Table 1. We divided the patients into two groups: one with patients who had received a genetic diagnosis of a specific form of hereditary ataxia, and a group with those who had not. Statistical analysis of independent samples *t*-test was performed in SPSS to compare the means of both groups and showed a statistically significant difference for the means of “age” and “age at disease onset” variables between the two subgroups; the group of patients that received a genetic diagnosis was significantly younger and had an early disease onset compared to the other group. The test did not show a statistically significant difference between the means for disease duration and SARA score. When comparing the groups of males and females using the independent samples *t*-test method in SPSS, the *p*-value was not significant (> 0.05; Table 1). Responses to all the single items of the questionnaire are summarized in Supplemental Information. The most commonly reported symptoms at disease onset were “poor balance” (reported by *n* = 46, or 61.3%, of respondents) and “impaired gait” (*n* = 12; 16%). In addition to the above, common symptoms at the time of the questionnaire were “dysarthria” (*n* = 23; 30.7%), “poor coordination” (*n* = 23; 30.7%), “poor fine motor skills” (*n* = 22; 29.3%), and “impaired vision” (*n* = 17; 22.7%). In the questionnaire, most of the participants described the disease progress as “slow” (*n* = 66; 88%).

**Table 1:** Characteristics of study population

	Total sample (*n* = 75)		Male (*n* = 40)		Female (*n* = 35)		With genetic diagnosis (*n* = 28)		Without genetic diagnosis (*n* = 47)	
	M	SD	M	SD	M	SD	M	SD	M	SD
Age (years)	57.3	15.4	59.1	16.5	55.3	14.0	48.4	13.7	62.6	13.9
Age at disease onset (years)	39.5	19.5	41.1	19.3	37.6	20.1	28.6	17.5	45.9	17.9
Disease duration (years)	18.6	12.4	18.1	11.9	19.3	13.2	20.4	15.1	17.5	10.6
SARA score (points)	13.2	9.0	13.4	8.5	13.0	9.7	13.6	10.6	13.1	8.0

This table shows the characteristics of all patients who filled out the questionnaire. Statistical analysis of the data from the subgroups who had an established genetic diagnosis and those who did not have a genetic diagnosis at the time when the questionnaires were collected showed a significant difference in age and age at disease onset. Patients from the group with an established genetic diagnosis were significantly younger and had an earlier disease onset. There were no significant differences (*p* ≤ 0.05) between gender

*M*, mean; *SD*, standard deviation; *n*, total number; *SARA*, scale for the assessment and rating of ataxia

Only 18 (24%) of the participants were working full-time or part-time, and 28 (37.3%) received disability benefits or were unemployed; 12 (16%) were retired; however, 16 (21.3%) did not answer this question.

### Patients’ Limitations and What Helps Patients Feel Better

**Fig. 1:**
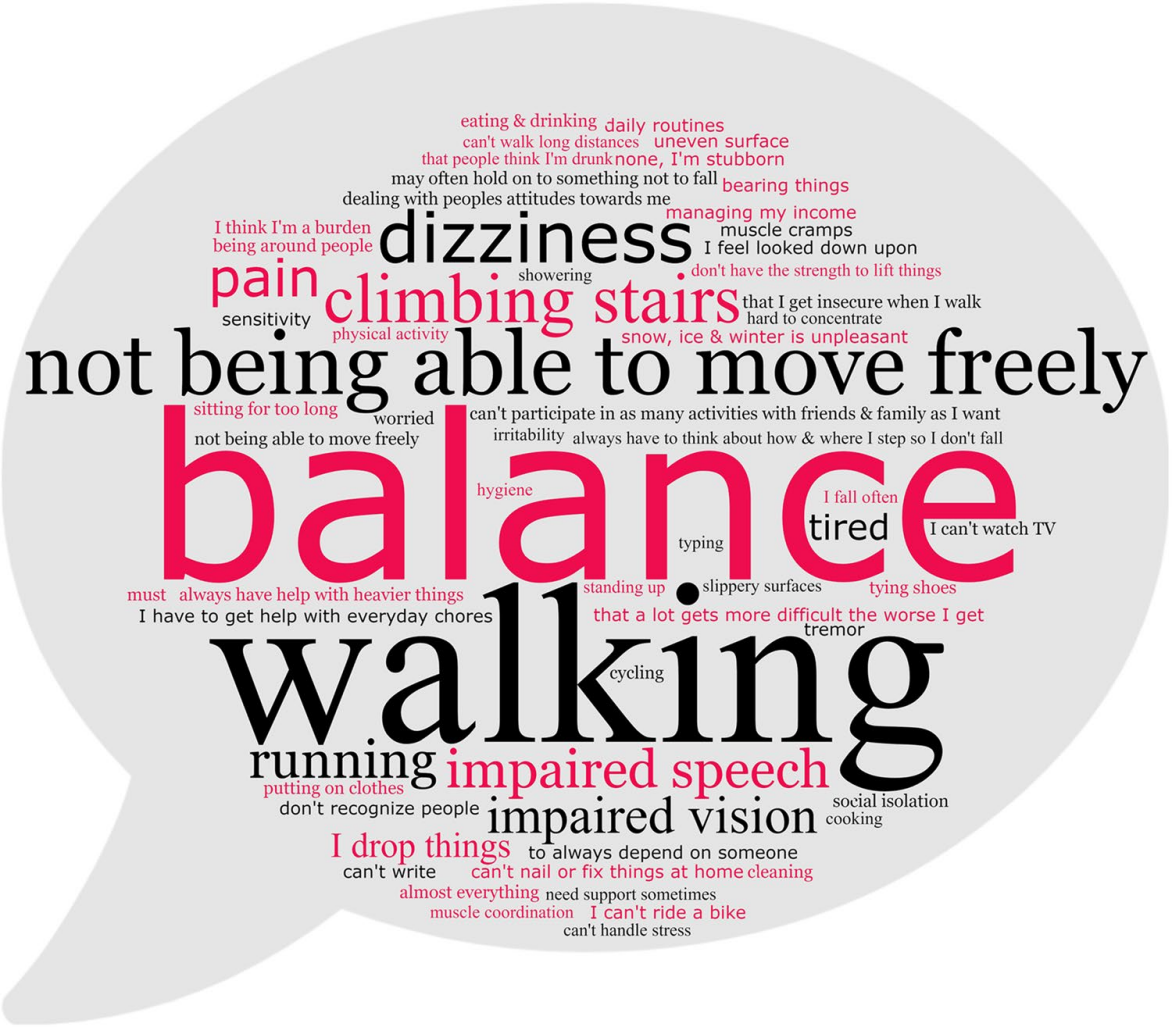
Ataxia patients’ perceived restrictions and difficulties in everyday life. Word cloud image of ataxia patients’ answers to the open question, “What is most difficult for you in your everyday life? What restrictions do you experience?” Forty-eight patients responded to these questions. Letter size corresponds with the number of patients who mentioned this topic, from 19 patients stating difficulties walking to 1 patient each for several topics shown in the smallest print. Image created with WordClouds.com

Limitations of balance (*n* = 29; 38.7%), mobility (*n* = 27; 36%), and coordination (*n* = 10; 13.3%) were the main difficulties that patients struggled with in everyday life. The detailed answers to the open questions are shown in Fig. 1. Some of the patients reported not being able to perform physical activities such as walking long distances (*n* = 18; 24%), cycling (*n* = 11; 14.7%), and playing sports (*n* = 8; 10.7%). Driving a car was possible for (*n* = 39; 52%).

Table 2 shows the patients’ responses to multiple-choice questions on the perceived effect of different therapeutic or supportive options. In total, less than half of the patients (*n* = 34; 45.3%) reported improvement or partial improvement, and 40% (*n* = 30) did not experience any improvement with any strategies; 10 patients did not answer this question.

**Table 2:** Different therapeutic or supportive options and experienced effect

What kind of treatment have you received?	*n*	%
Physiotherapy	44	58.7
Counseling	15	20.0
Speech therapy	16	21.3
Symptomatic treatment (medications)	19	25.3
Other	16	21.3
Have you noticed any improvement?	*n*	%
Yes	6	8.0
Partly	28	37.3
No	30	40.0

All 75 respondents had the possibility to choose multiple answers to the questions above. *n* = total number of answers; % = the percentage of answers from the total of 75 patients with ataxia

**Fig. 2:**
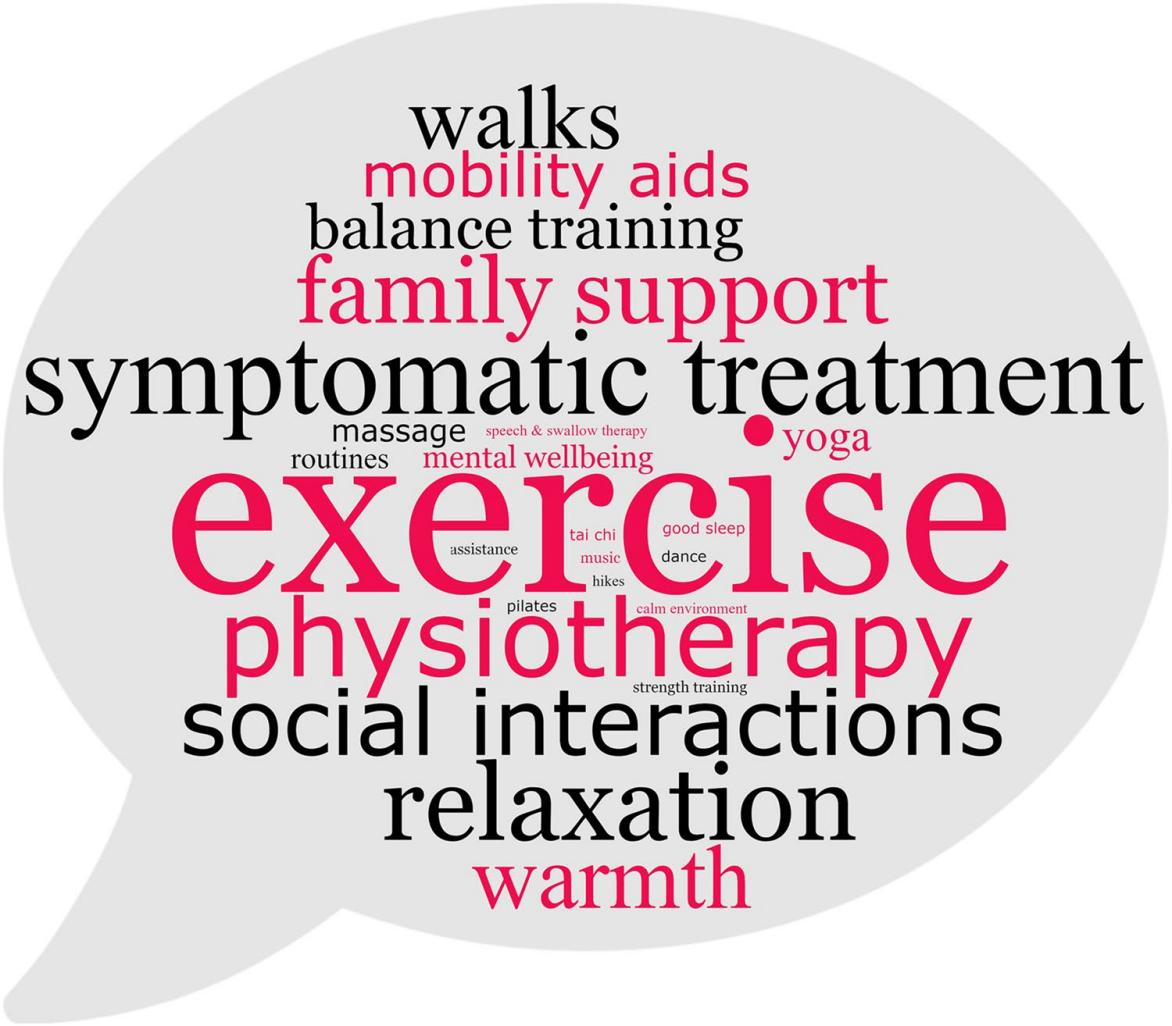
Ataxia patients’ answers to “What gives results or helps you feel better?” Word cloud image of ataxia patients’ answers to the open question, “What gives results or helps you feel better?” Fifty-five patients responded to this question. Letter size corresponds with the number of patients who mentioned this topic, from 19 patients stating exercise to 1 patient each for several topics shown in the smallest print. Image created with WordClouds.com

Figure 2 shows patients’ replies to the open-ended question on what led to improvement of symptoms or overall well-being. Most patients reported that exercise or physical activity were helpful, and they also mentioned spending time with family, stress-reducing and relaxing measures, mobility aids, and exposure to warm temperature, among others, had an effect.

### Genetic Testing, Obtaining Disease-Related Information and Support

Table 3 presents the results of questions about genetic testing, diagnosis-related information, and whom patients turn to for moral support.

**Table 3:** Questionnaire results on diagnosis-related information

Questionnaire item	*n*	%
Have you undergone genetic testing and been informed of the results?		
Yes, by a neurologist	33	44.0
Yes, by a clinical geneticist	10	13.3
I don’t know	3	4.0
Have you received a genetic diagnosis?		
Yes	28	37.3
No	47	62.7
Do you feel that you have received enough information about your disease?		
Yes	21	28.0
Partly	30	40.0
No	24	32.0
Where/from whom did you receive the most relevant information about your disease?		
Doctor	50	66.7
Family members with the same disease	8	10.7
Internet	9	12.0
Patient organization	3	4.0
How do you usually seek information about your disease?		
Talk to my doctor	42	56.0
Articles/journals	9	12.0
From individuals in the same situation (internet forums/blogs)	14	18.7
Membership in a neurological patient organization	13	17.3
Other/internet	30	40.0
Who do you turn to if you want to talk about your disease?		
Close family	59	78.7
Close friends	22	29.3
Acquaintances	6	8.0
Healthcare professionals	15	20.0
No one	10	13.3

Questions about genetic testing, access to diagnosis-related information and communication support are presented in the table above. *n* = number of answers for each question; % = percentage of all 75 respondents

Approximately one-third of the patients, 32.0% (*n* = 24), answered that they felt insufficiently informed about their ataxia. They report that they usually seek information about their disease by talking to their doctor 56.0% (*n* = 42) or by searching on the internet 40.0% (*n* = 30). The majority 66.7% (*n* = 50) stated they had obtained the most relevant information from their doctor. Patients turn to close family 78.7% (*n* = 59), close friends 29.3% (*n* = 22), and healthcare professionals 20.0% (*n* = 15) for discussion of disease-related questions and support.

Of all participants, 57 answered that they had children; of these, 54 were worried that the disease will be carried on to the next generations, and 2 had adopted children. Of all included patients, 57.3% (*n* = 43) reported that they had undergone genetic testing to elucidate the exact type of their ataxia and had been informed about the results prior to their inclusion in our study. Twenty-eight (37.3%) had received a genetic diagnosis, and 47 (62.7%) did not have a genetic diagnosis at the time of the collection of questionnaires. The two largest groups in our cohort were SCA3 and SCA2 with complex ataxias; almost all patients had additional non-cerebellar signs and symptoms, and all the genetically verified forms were complex ataxia forms. Supplemental Table 1 lists the genetic diagnoses in our series.

**Fig. 3:**
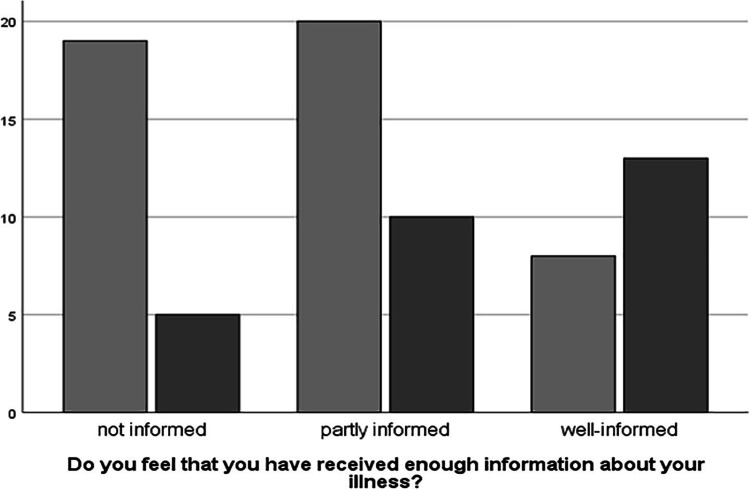
Having a genetic diagnosis and the degree of feeling informed. Patients were divided in two different groups as follows: one group had an established genetic diagnosis (dark gray bars), and the other group did not (light gray bars). Patients in both groups were asked how well-informed they felt about their disease (“well-informed,” “partly well-informed,” and “not well-informed”). Patients who had received a genetic diagnosis felt better informed than those who had not (*p* = 0.015). *Y*-axis signifies the number of responses

Among the 28 patients who had a genetic diagnosis, 82.1% (*n* = 23) felt “well-informed” or “partly well-informed,” compared to 59.6% (*n* = 28) of the patients who did not have a confirmed genetic diagnosis (*n* = 47) Fig. 3*.* Pearson chi-square test showed a significant association between having a genetic diagnosis and feeling well-informed (*X*^2^(2) = 8.417, *p* = 0.015).

### Quality of Life

Table 4 summarizes the results of the EQ-5D-3L questionnaire for the ataxia patients divided into age categories. Most of the patients were affected by the disease to some degree in all five EQ-5D-3L dimensions. Self-care and usual activities were the most problematic areas for many of our patients. A relatively large group of patients did not answer the question about mobility (*n* = 20). We explored if this is associated with a higher SARA score in those individuals. An independent samples *t*-test was performed and showed that the group who did not answer indeed had a much higher SARA mean (22.4 ± 9.1) than the group who answered the question (9.6 ± 6.4), the mean difference between the groups was statistically significant: 12.8 (*p*-value < 0.005, 95% CI [9.06–16.6]).

**Table 4:** EQ-5D-3L dimensions in patients with hereditary ataxia by age group

EQ-5D-3L dimension		Age groups (years)													
		18–24		25–34		35–44		45–54		55–64		65–74		75 +	
		*N* = 2		*N* = 4		*N* = 12		*N* = 13		*N* = 16		*N* = 18		*N* = 10	
		*n*	%	*n*	%	*n*	%	*n*	%	*n*	%	*n*	%	*n*	%
Mobility	No problems	1	50.0	1	25.0	1	8.3	1	7.7	2	12.5	1	5.6	0	0.0
	Some problems	1	50.0	2	50.0	10	83.3	8	61.5	8	50.0	12	66.7	7	70.0
	Confined to bed	0	0.0	0	0.0	0	0.0	0	0.0	0	0.0	0	0.0	0	0.0
Self-care	No problems	1	50.0	3	75.0	11	91.7	9	69.2	9	56.3	11	61.1	4	40.0
	Some problems	0	0.0	1	25.0	1	8.3	2	15.4	2	12.5	7	38.9	5	50.0
	Unable to	1	50.0	0	0.0	0	0.0	2	15.4	5	31.3	0	0.0	1	10.0
Usual activities	No problems	1	50.0	2	50.0	7	28.3	3	23.1	3	18.8	3	16.7	6	60.0
	Some problems	0	0.0	1	25.0	4	33.3	7	53.8	6	37.5	11	61.1	0	0.0
	Unable to	1	50.0	1	25.0	1	8.3	2	15.4	7	43.8	3	16.7	3	30.0
Pain/discomfort	No	0	0.0	1	25.0	5	41.7	4	30.8	2	12.5	4	22.2	8	80.0
	Some	2	100.0	2	50.0	7	28.3	6	46.2	10	62.5	13	72.2	2	20.0
	Extreme	0	0.0	1	25.0	0	0.0	3	23.1	2	12.5	1	5.6	0	0.0
Anxiety/depression	No	0	0.0	1	25.0	6	50.0	3	23.1	4	25.0	7	38.9	5	50.0
	Some	2	100.0	2	50.0	5	41.7	9	69.2	10	62.5	10	55.6	4	40.0
	Extreme	0	0.0	1	25.0	1	8.3	1	7.7	2	12.5	0	0.0	0	0.0

Not all patients responded to all the questions; for the first measured dimension “mobility” 20 patients did not answer, 3 for “usual activities,” 2 for “pain/discomfort,” 2 for “anxiety/depression”; all patients answered the “self-care” question. Most of the responders were 35 years or older. *N* = number of responders for each age group, *n* = number of responders for each answer alternative, and % = percent of responders within the age group

Table 5 are presented the results from the Mann–Whitney comparison analysis in SPSS between the normal population and patients with ataxia. The test results show that the quality of life in patients with ataxia is statistically more affected for almost all age groups and in all five domains of EQ-5D-3L compared to the normal population. No statistically significant difference could be seen for pain and discomfort in the age group of 65 years and older.

**Table 5:** Comparing EQ-5D-3L results between ataxia patients and normal population

EQ-5D-3L dimension	Age group (years)		*N*	Mean rank	*P*-value
Mobility (no problems = 0; some problems = 1; confined to bed = 2)	18–44	General population	254	129.22	
		Ataxia patients	16	235.19	
		Total	270		0.000
	45–64	General population	171	88.44	
		Ataxia patients	19	159.00	
		Total	190		0.000
	65 and above	General population	97	51.66	
		Ataxia patients	20	94.58	
		Total	117		0.000
Self-care (no problems = 0; some problems = 1; unable to = 2)	18–44	General population	254	135.03	
		Ataxia patients	18	157.19	
		Total	272		0.000
	45–64	General population	171	95.19	
		Ataxia patients	29	131.79	
		Total	200		0.000
	65 and above	General population	97	57.06	
		Ataxia patients	28	83.59	
		Total	125		0.000
Usual activities (no problems = 0; some problems = 1; unable = 2)	18–44	General population	254	132.87	
		Ataxia patients	18	187.69	
		Total	272		0.000
	45–64	General population	171	90.56	
		Ataxia patients	28	157.64	
		Total	199		0.000
	65 and above	General population	96	54.22	
		Ataxia patients	26	88.38	
		Total	122		0.000
Pain/discomfort (no = 0; some = 1; extreme = 2)	18–44	General population	254	133.17	
		Ataxia patients	18	183.53	
		Total	272		0.001
	45–64	General population	171	94.57	
		Ataxia patients	27	130.70	
		Total	198		0.001
	65 and above	General population	97	62.82	
		Ataxia patients	28	63.63	
		Total	125		0.905
Anxiety/depression (no = 0; some = 1; extreme = 2)	18–44	General population	254	133.12	
		Ataxia patients	18	184.14	
		Total	272		0.001
	45–64	General population	170	93.02	
		Ataxia patients	29	140.93	
		Total	199		0.000
	65 and above	General population	95	57.02	
		Ataxia patients	26	75.54	
		Total	121		0.002

We compared our data with published EQ-5D-3L data from the Swedish population from individuals selected randomly from the nationwide address register [13]. We used Mann–Whitney comparison analysis in SPSS. Because of the multiple comparisons between the population groups, the *p*-value was adjusted according to the Bonferroni method; the corrected *p*-value is 0.01. The test results show that the quality of life in patients with ataxia is statistically more affected for all but one age group and in all five domains of EQ-5D-3L compared to the normal population. No statistically significant difference was seen for pain and discomfort in the age group of 65 years and older. *N* = number; mean rank = the average of the ranks for all observations within each sample. The higher the mean rank value, the further a group’s average rank is from the average rank of ataxia patients and the general public within the same age group combined

## Discussion

In this questionnaire-based study, patients with various types of hereditary ataxias reported disabilities that impaired their daily lives. Patients had a significantly lower overall quality of life compared to age-matched population data [12]. Our study recorded patients’ perceptions of the effect of symptomatic and supportive therapies and coping strategies, where physiotherapy and close family members’ or friends’ support were important for patients’ overall well-being. It showed that patients used various sources to obtain information about their diseases but considered personal information provided by their doctors the most reliable. Patients with a verified genetic diagnosis had a lower average age at onset and felt more well-informed about their disease than patients without a genetic diagnosis.

In our study, the majority of patients described the progression of their disease as slow. Some of the most challenging problems appeared to be mobility, coordination, and balance. Therefore, activities such as long walks, cycling, or playing sports were usually avoided or have become impossible. The majority of patients received disability benefits or were only able to work part-time, which we assume is the direct result of the disability caused by the disease. About half of the participants reported that various forms of treatment, including symptom-alleviating medications, physiotherapy, counseling, or speech therapy, had a clear or partial effect.

Only a few previous studies investigated aspects of quality of life in patients with ataxia. Schmitz-Hübsch et al. [6] presented the variability and predictors of subjective health in a multi-center series of 526 patients with SCA1, SCA2, SCA3, or SCA6. As in our study, subjective health status was assessed using a form of the EQ-5D (Euroqol) questionnaire. Patient-reported quality of life was compromised for all five domains of these autosomal dominant ataxia forms in the questionnaire (mobility, usual activities, pain/discomfort, depression/anxiety, and self-care). Authors delineated differences between these SCA forms, which we were not able to do because our series had a wider variety of genetic and nongenetic forms of ataxia with lower numbers of patients for each form. Xiong et al. [7] assessed health-related quality of life in 651 individuals with Friedreich ataxia using a generic self-administered questionnaire SF-36, that measures physical and mental health, and symptom-specific scales examining vision, fatigue, pain, and bladder function. The study showed that the physical domains affect the health-related quality of life most, from the patient perspective, similar to our study. D’Ambrosio et al. [8] studied the disability and quality of life scores in 151 patients with both Friedreich ataxia and other hereditary ataxias. They designed a broad questionnaire based on the clinical experience of the authors and suggestions from patients and their relatives. Walking was the most affected of all activities. Intellectual or cognitive dysfunction was common in other hereditary ataxias but not in Friedreich ataxia. The authors concluded that, despite their severe disability, ataxia patients managed to maintain a satisfactory quality of life. Social and family support was important to keep a low dependence, which patients in our study also highlighted. Joyce et al. [14] investigated how living with hereditary ataxia affected the quality of life in patients and, in contrast to our study, also asked their caregivers. The authors created a semi-structured interview model covering motor, cognitive, and psychosocial activities. Fifty-five patients with a diagnosis of hereditary ataxia or an unknown cause for ataxia and 47 caregivers were included. Both groups reported that posture/gait, speech speed, and precision and daily activities/fine motor tasks were the most affected domains. Patients and caregivers agreed in most of their responses, with the exception that patients rated their ability to switch attention better and their communication skills worse than their caregivers did.

Our study differs in part from these previous studies on the quality of life in ataxia patients. It has a broader representation of different ataxia diagnoses based on patients recruited from among all ataxia patients at our center. It included early- and late-onset patients and various forms of ataxia as encountered in our population. Although we also included patients that were referred through a patient organization, we consider the patients studied here to represent our overall ataxia population relatively well. The present study adds a comparison of the quality-of-life reports between patients with ataxia and published data from a randomly selected group from the general population from the same country as a way to mirror the differences between patients with disabilities and the society they live in. While we are not aware of previous similar studies on ataxia patients, the quality of life in patients with other neurological diseases similarly was lower than in the general population [15–17].

Our study also adds knowledge on ataxia patients’ perceived need for information about their disease and how they obtain this information, which, to our knowledge, has not previously been studied. Previous studies on patients with neurodegenerative disorders such as dementia concluded that available sources of information were extensive, but more emphasis needs to be placed on healthcare service-related information [18]. In our study, most patients experienced that they obtained the most relevant information about their disease by talking to their doctor, but internet search appeared as an alternative as well. Similar to our results, health professionals were the most trusted source of information for caregivers of individuals with spinal cord injury, and health education supported caregivers in their activities, reduced subjective burden, and improved their health [19]. Previous work described how the internet era made it accessible for patients to learn and interact with peers online but also studied concerns around safety and misinformation [20, 21]. Despite the increasing availability of general information sources including the internet, there is a clear need to continue to provide individual information and support for patients with hereditary ataxia, since healthcare professionals are the most trusted sources according to our survey.

We found that patients with a genetic diagnosis were younger and had an earlier disease onset compared to those without a genetic diagnosis. This might be explained by the fact that many forms of hereditary ataxias with late onset still have an unknown genetic cause and are usually diagnosed as idiopathic ataxia. It may also reflect the fact that young or young-onset patients more often than older- or later-onset patients were tested genetically in clinical practice. We currently reanalyze all patients without a genetic diagnosis in our cohort with whole exome or whole genome sequencing and plan to publicize our findings independently.

We also found a significant association between having received a genetic diagnosis and feeling more well-informed about the disease. This supports the usefulness of receiving a genetically verified diagnosis of the exact ataxia subtype rather than a generic diagnosis of “hereditary ataxia.” However, most patients in our study population were worried that the disease would be passed on to future generations, and this also included patients with the recessive disease without a consanguineous spouse. This highlights the importance to offer genetic counseling to each family and to making sure that patients and families understand and remember the possible risk (or relative lack thereof) that their offspring will be affected by the same disease.

Our study is limited by a relatively modest number of respondents and by a relative percentage of patients who were contacted but who did not agree to participate or who did not return their questionnaires. An increased SARA score was associated with a lower rate of response for the mobility section of the EQ-5D questionnaire, even among those patients who participated in our study. This supports the hypothesis that these patients are more affected by their disease and may have difficulty reporting it. Patients who declined to participate in our research study or who did not return the questionnaire may have alternative diagnoses, may not be interested in research as they have only very mild symptoms or may be so severely disabled that they were unable to take part. However, the study population consisted of patients recruited for a research study from a variety of different sources, which provides a better representation of different ataxia diagnoses. Culture-specific and societal factors likely influence patient experience. Nevertheless, we consider our sample of patients relatively representative of a large part of ataxia patients in neurology clinics in many countries.

## Conclusion

We hope that our study’s results on hereditary ataxia patients’ perspective can be employed to improve how health services investigate and provide disease-related information to and support these patients. In particular, our findings suggest that patients should be recommended physical activity, including individual exercise, physical leisure activities, and physio- or ergotherapy, because many ataxia patients experience that this makes them feel better. Another recommendation is to encourage patients to maintain close contact with family and near friends. Healthcare providers can improve patients’ well-being by providing medical information about the disease.

### Supplementary Information

Below is the link to the electronic supplementary material.

Supplementary file1 (PDF 114 KB)

Supplementary file2 (PDF 193 KB)

Supplementary file3 (PDF 85 KB)

## Data Availability

All data generated or analyzed during this study are included in this published article and its supplementary information files.

## Materials Availability

All data generated or analyzed during this study are included in this published article and its supplementary information files.
