# Supplementary material for: Patients’ Perspective in Hereditary Ataxia
Source: Cerebellum. 2022 Dec 16;23(1):82–91. doi: 10.1007/s12311-022-01505-1 (PMC10864479; doi:10.1007/s12311-022-01505-1)
Supplement: Supplementary file 1 — Supplementary file1 (PDF 114 KB) [file 12311_2022_1505_MOESM1_ESM.pdf]

Skånes universitetssjukhus
En del av Region Skåne

Ataxia Project

How old are you?_______________________________

How old were you when you got your first symptoms? And which were they?

____

____

Briefly describe your current symptoms:

____

____

____

____

____

How do you experience the course of the disease?

◯ Fast (within days, months, one year)

◯ Slow (within a few years)

Have you been genetically tested and informed about the results?

◯ Yes, by a specialist in neurology

◯ Yes, by a specialist in genetics

◯ No

Do you feel that you have got enough information about your disease?

◯ Yes

◯ No

◯ Partly

From where/ by whom did you get the most relevant information?

____

____

____

How do you usually seek information about your disease?

◯ Talk to a doctor

◯ From articles/papers

◯ From other people in the same situation

◯ I am a member of a neurological association

◯ Other:____

What kind of treatment have you received?

◯ Physiotherapy

◯ Counselling/Psychological support

◯ Speech and swallowing therapy by a speech therapist

◯ Alleviating medicine: ____

◯ Other treatments: ____

Have you noticed any improvement after treatment?

◯ Yes

◯ No

◯ Partly

What gives results or help you feel better?

____

____

____

____

How can the current treatment options be improved?

____

____

____

____

What is most difficult for you in your everyday life? What restrictions do you experience?

____

____

____

____

____

Are there any activities that you do not manage or avoid? If yes, which are they?

____

____

____

____

____

How is your mobility?

◯ I´m, walking without difficulty on;

- **a)** Uneven ground
- **b)** Even ground

◯ I´m walking with some difficulty;

- **a)** I use support in the environment
- **b)** I use a walking cane/stick
- **c)** I use a walker

◯ I use a wheelchair

◯ I´m bedridden

How is your speech?

◯ Normal

◯ Sometimes affected

◯ Most often affected, I often have to repeat words

◯ It´s hard to understand

How do you manage your daily hygiene?

- ◯ I don´t need any help with my daily hygiene, food or clothing
- ◯ I have some problem washing or dressing myself
- ◯ I am unable to wash or dress myself

Usual activities (e.g. work, study, housework, family or leisure activities)

- ◯ I have no problems with performing my usual activities
- ◯ I have some problems with performing my usual activities
- ◯ I am unable to perform my usual activities

Pain/discomfort

- ◯ I have no pain or discomfort
- ◯ I have moderate pain or discomfort
- ◯ I have extreme pain or discomfort

Anxiety/depression

- ◯ I am not anxious or depressed
- ◯ I am moderately anxious or depressed
- ◯ I am extremely anxious or depressed

Who do you turn to if you want to talk about your disease?

- ◯ Family
- ◯ Close friends
- ◯ Acquaint
- ◯ Healthcare professionals
- ◯ I do not talk to anyone

Do you drive?

- ◯ Yes
- ◯ No
- ◯ I have access to a travel service (taxi service for disabled)

Do you work?

- ◯ Full-time
- ◯ Part time
- ◯ Sick leave
- ◯ Unemployed

Do you have children?

- ◯ Yes
- ◯ No

Are you worried that the disease will be carried on to the next generation?

- ◯ Yes
- ◯ No

What do you expect from the future research on ataxia?

____

____

____

____

____

Do you have any comments or suggestions for improvements?

____

____

____

____

____
