# Supplementary material for: Patients’ Perspective in Hereditary Ataxia
Source: Cerebellum. 2022 Dec 16;23(1):82–91. doi: 10.1007/s12311-022-01505-1 (PMC10864479; doi:10.1007/s12311-022-01505-1)
Supplement: Supplementary file 2 — Supplementary file2 (PDF 193 KB) [file 12311_2022_1505_MOESM2_ESM.pdf]

Patient Perspective in Hereditary Ataxia

Sorina Gorcenco MD, Christin Karremo RN, Andreas Puschmann MD PhD

Supplemental material 2: Summary of responses to all the single items of the questionnaire

		N	% of total sample (n=75)
**Sex**	female	35	46,7%
	male	40	53,3%
**How do you experience the course of the disease?**	fast	8	10,7%
	slow	66	88,0%
**Have you undergone genetic testing and been informed of the results?**	yes by a neurologist	33	44,0%
	yes by a geneticist	10	13,3%
	I don't know	3	4,0%
	no	29	38,7%
**Do you feel that you have received enough information about your**	yes	21	28,0%
	partly	30	40,0%
	no	24	32,0%
**Where/from whom did you receive the most relevant information?**	neurologist/ doctor	50	66,7%
	family	8	10,7%
	internet	9	12,0%
	patient organization	3	4,0%
**How do you usually seek information about your illness?**	talk to my doctor	42	56,0%
	articles/journals	9	12,0%
	forums/individuals in the same situation	14	18,7%
	membership in a neurological organisatio	13	17,3%
	other/internet	30	40,0%
**Who do you turn to if you want to talk about your disease?**	close family	59	78,7%
	close friends	22	29,3%
	acquaintances	6	8,0%
	healthcare professionals	15	20,0%
	no one	10	13,3%
**Do you have children?**	yes	57	76,0%
	no	18	24,0%
**Are you worried that the disease will be carried on to the next generation?**	yes	55	73,3%
	no	17	22,7%
	didn't answer	3	4,0%
**Symptoms at onset**	poor balance	46	61,3%
	gait impairment	12	16,0%
	poor coordination	5	6,7%
	dizziness	6	8,0%
	poor fine motor skills	5	6,7%
	tremor	5	6,7%
	muscle weakness	3	4,0%
	polyneuropathy	2	2,7%
	forgetfulness	2	2,7%
	fatigue	1	1,3%
	poor concetration	1	1,3%
	hearing loss	1	1,3%
	dysarthria	1	1,3%
	spasticity	1	1,3%

	double vision	1	1,3%
Current symptoms	poor balance	50	66,7%
	gait impairment	22	29,3%
	dysarthria	23	30,7%
	poor coordination	23	30,7%
	poor fine motor skills	22	29,3%
	impaired vision	17	22,7%
	fatigue	13	17,3%
	muscle weakness	12	16,0%
	dizziness	12	16,0%
	spasticity	12	16,0%
	polyneuropathy	10	13,3%
	pain	10	13,3%
	tremor	9	12,0%
	dysfagia	7	9,3%
	muscular tension	5	6,7%
	incontinence	4	5,3%
	insomnia	3	4,0%
	anxiety	3	4,0%
	depression	3	4,0%
	hearing loss	2	2,7%
	forgetfulness	2	2,7%
	hallucinations	1	1,3%
	poor concetration	1	1,3%
	foot deformity	1	1,3%
	skin changes	1	1,3%
	constipation	1	1,3%
	weight loss	1	1,3%
What kind of treatment have you received?	physiotherapy	44	58,7%
	counseling	15	20,0%
	speech therapy	16	21,3%
	symptomatic treatment	19	25,3%
	other	16	21,3%
Have you noticed any improvement after treatment?	yes	6	8,0%
	partly	28	37,3%
	no	30	40,0%
What gives results or helps you feel better?	exercise	19	25,3%
	symptomatic treatment	8	10,7%
	physiotherapy	7	9,3%
	relaxation	7	9,3%
	warmth	6	8,0%
	social interactions	6	8,0%
	family support	6	8,0%
	walks	6	8,0%
	mobility aids	4	5,3%
	balance training	4	5,3%
	yoga	3	4,0%
	massage	2	2,7%
	mental wellbeing	2	2,7%
	routines	2	2,7%

	tai chi	1	1,3%
	strength training	1	1,3%
	speech & swallow therapy	1	1,3%
	pilates	1	1,3%
	music	1	1,3%
	hikes	1	1,3%
	good sleep	1	1,3%
	assistance	1	1,3%
	calm environment	1	1,3%
	dance	1	1,3%
**What is most difficult for you in your everyday life? What restrictions do you experience?**	walking	19	25,3%
	balance	18	24,0%
	not being able to move freely	7	9,3%
	climbing stairs	5	6,7%
	dizziness	5	6,7%
	impaired speech	4	5,3%
	impaired vision	4	5,3%
	pain	4	5,3%
	running	4	5,3%
	I drop things	3	4,0%
	tired	3	4,0%
	almost everything	2	2,7%
	always have to think about how & where	2	2,7%
	bearing things	2	2,7%
	cycling	2	2,7%
	hygiene	2	2,7%
	I have to get help with everyday chores	2	2,7%
	putting on clothes	2	2,7%
	showering	2	2,7%
	snow, ice & winter is unpleasant	2	2,7%
	social isolation	2	2,7%
	standing up	2	2,7%
	to always depend on someone	2	2,7%
	being around people	1	1,3%
	can't handle stress	1	1,3%
	can't nail or fix things at home	1	1,3%
	can't participate in as many activities wit	1	1,3%
	can't walk long distances	1	1,3%
	can't write	1	1,3%
	cleaning	1	1,3%
	cooking	1	1,3%
	daily routines	1	1,3%
	dealing with peoples attitudes towards m	1	1,3%
	don't have the strength to lift things	1	1,3%
	don't recognize people	1	1,3%
	eating & drinking	1	1,3%
	hard to concentrate	1	1,3%
	I can't ride a bike	1	1,3%
	I can't watch TV	1	1,3%
	I fall often	1	1,3%

	I feel looked down upon	1	1,3%
	I think I'm a burden	1	1,3%
	irritability	1	1,3%
	managing my income	1	1,3%
	may often hold on to something not to fa	1	1,3%
	muscle coordination	1	1,3%
	muscle cramps	1	1,3%
	must always have help with heavier thing	1	1,3%
	need support sometimes	1	1,3%
	none, I'm stubborn	1	1,3%
	not being able to move freely	1	1,3%
	physical activity	1	1,3%
	sensitivity	1	1,3%
	sitting for too long	1	1,3%
	slippery surfaces	1	1,3%
	that a lot gets more difficult the worse I g	1	1,3%
	that I get insecure when I walk	1	1,3%
	that people think I'm drunk	1	1,3%
	tremor	1	1,3%
	tying shoes	1	1,3%
	typing	1	1,3%
	uneven surface	1	1,3%
	worried	1	1,3%
**Are there any activities that you do not manage or avoid? If yes, which are they?**	Walking long distances	18	24,0%
	Running	7	9,3%
	Jumping	1	1,3%
	Cycling	13	17,3%
	Driving	6	8,0%
	Climbing	3	4,0%
	Crowds(shops, nightclubs, pubs, restaura	7	9,3%
	Cooking	2	2,7%
	writing	3	4,0%
	speaking	2	2,7%
	singing	1	1,3%
	dancing	1	1,3%
	selfcare	4	5,3%
	clean	1	1,3%
	exercise	3	4,0%
	playing sports	8	10,7%
	travelling	1	1,3%
	play computer games	1	1,3%
**Do you drive?**	yes	39	52,0%
	no	20	26,7%
	I have travel service	3	4,0%
**Do you work?**	full-time	6	8,0%
	part-time	13	17,3%
	sick leave	25	33,3%
	unemployed	3	4,0%
	retired	12	16,0%
	didn't answer	16	21,3%

**What do you expect from the future research on ataxia?**	a cure/a drug to slow down the disease	43	57,3%
	finding the cause	11	14,7%
	more information about the disease	11	14,7%
	continuing research	4	5,3%
	to get a diagnosis	6	8,0%
