# Supplementary material for: Patients’ Perspective in Hereditary Ataxia
Source: Cerebellum. 2022 Dec 16;23(1):82–91. doi: 10.1007/s12311-022-01505-1 (PMC10864479; doi:10.1007/s12311-022-01505-1)
Supplement: Supplementary file 3 — Supplementary file3 (PDF 85 KB) [file 12311_2022_1505_MOESM3_ESM.pdf]

Supplemental Table1

Genetic diagnosis	Number of participants who stated they had a genetic diagnosis in the questionnaire	Disease duration (range, years)
**SCA3** (dom)	10	5 – 30
**SCA2** (dom)	4	2 – 15
**ATXPC** (dom)	4	10 – 46
**EA2** (dom)	2	30, 52
**EA1** (dom)	1	47
**SCA17** (dom)	1	5
**AT** (rec)	2	40, 46
**FRDA** (rec)	2	10, 12
**CANVAS** (rec)	1	7
**BVVL2** (rec)	1	33

The table illustrates the different monogenic forms of ataxia in 28 patients that reported to have a genetic diagnosis at the time of the study.
Within these groups, there was a wide range of disease duration.
SCA3 – spinocerebellar ataxia type 3; SCA2 – spinocerebellar ataxia type 2; ATXPC – ataxia pancytopenia syndrome; EA2 – episodic ataxia type 2; EA1 – episodic ataxia type 1; SCA17- spinocerebellar ataxia type 17; AT – ataxia telangiectasia; FRDA – Friedreich's ataxia; CANVAS – cerebellar ataxia with neuropathy and vestibular areflexia syndrome; BVVL2 – Brown-Vialetto-Van-Laere syndrome; dom – autosomal dominant mode of inheritance; rec – autosomal recessive mode of inheritance.
